# Editorial

**Published:** 1955-09

**Authors:** 


### *Editorial*

The last number of this *Journal* appeared in October 1954. Mr. H. Keith Lucas has bravely tackled the task of editorship for four years. But he has had less and less time for this and, regretfully, he has now resigned. The Medico-Chirurgical Society owes him much, for without his help this journal would have perished four years ago. And without its *Journal* the Society would have lost one of its proudest features. In its new form the Medical Journal of the South-West has served to bring together the medical societies and clubs scattered through the south-west region. Most of the credit for this improvement also is due to the vision and energy of Mr. Lucas.

The articles published in this *Journal* fall into two broad groups. There are those papers which have already been read before this Society or before other Societies in this region. There is plenty of this matter awaiting publication in future numbers. Secondly there are original papers by younger men—often registrars—who report cases or describe their own work. The supply of papers of this group is low, and contributions of this nature will be welcome.

